# Supplementary material for: Understanding the experience of veterans who require lower limb amputation in the veterans health administration
Source: PLoS One. 2022 Mar 18;17(3):e0265620. doi: 10.1371/journal.pone.0265620 (PMC8932557; doi:10.1371/journal.pone.0265620)
Supplement: S1 File — (DOCX) [file pone.0265620.s001.docx]

**Veteran Dysvascular Amputee Interview Guide**

**Interviewer Name:**

**Date:**

**Time Start:**

**Time End:**

Hello [Mr./Ms. interview participant name],

My name is [interviewer name], and I am a researcher at the VA Medical Center in Seattle, Washington. I am calling *(choose appropriate one*):

- [to follow up on a letter we sent you about a research study we are conducting. We did not yet hear back from you, so we wanted to follow up with a phone call.] OR
- [to return a call from you about the letter you received from us.]

Is this a good time to talk?

- *If yes, continue.*
- *If no, arrange a time to call back.*

As part of this VA-sponsored research study, we are interviewing veterans who have had a mid-foot or below knee amputation. We are trying to understand the process that led to the decision to have an amputation. For example, we would like to know if there was information you wish you had that perhaps you didn’t, and how the process could be improved. If you participate in this study, you would receive $40 payment in the mail for your participation. Are you interested in participating in this study?

- *[If no] thank them for their time and end the screening.*
- *[If yes**]* I want to provide you with some additional information about participation. As well as interview you. This process should take 50-60 minutes. Do you have time now, or should I call back?
  - *If yes, continue.*
  - *If no, arrange a time to call back.*
  - Do you have a copy of the Information Statement that was mailed to you? *l*
- *If eligible participants do not have their copy of the information statement readily available ask for their permission to email a copy to them. If they agree, email the Information Statement to them and proceed with the consent discussion and interview after they receive it. If they decline to have it emailed to them we will mail them another copy and reschedule the interview.]*

*[Conduct informed consent discussion, while referencing the Information Statement.]*

You can stop the interview at any time, and let me know if you’d rather not answer a particular question.

Do you have any questions?

**Shared Decision Making Veteran Patient Interview Guide-I**

*In order to make sure we capture all of the information you give us in the questions I am about to ask, we would like to record this interview. Only members of the study team will have access to your responses. As a reminder, you can skip any questions you do not wish to answer, and you can let me know at any point if you’d like to stop the interview.*

*Are you ok with me recording this interview?*

[If yes, start recording]: *Okay, to confirm, I’m starting the recording.*

[If no]: *Would you like to continue the interview and I will take notes?*

**CONSENT TO AUDIO-RECORD THE PHONE INTERVIEW**

**PARTICIPANT CONSENTED FOR AUDIO-RECORDING: YES NO**

**CONSENT OBTAINED BY:**

_______________________________ ________________________

Signature Print

**DATE CONSENT OBTAINED:** ____________________________

[When recording has started]:  *I have started the recorder, please confirm that you gave us your permission to record the interview.*

If participant changes his/her mind, stop recording.

If permission confirmed, continue.

Grounded prompts: If responses are limited or require clarification, probes may be used to elicit more detailed responses. Probes should use words or phrases presented by the participant using one of the following formats:

- *What do you mean by* ____________?
- *Tell me more about* ____________.
- *Give me an example of* ____________.
- *Tell me about a time when* ____________.
- Who ____________?
- Where ____________?
- When____________?
- Are there more examples of ________?

*We would like to learn about your experience related to your amputation. This information will help us to improve how we provide care to other Veterans going through this process.* ***Please know that there are no right or wrong answers to any of our questions.***

1. *Tell me about your amputation experience.*
2. *Tell me about how this all started.*
3. *Tell me about any conversations you had about amputation before the amputation.*
   1. *[If Needed] Tell me about any conversations you had with your doctor about amputation before the amputation.*
4. *Is there anything else you would like to share with us?*
5. *Do you have any questions for us?*

Do you have any questions for us, or is there anything else you would like to tell us?

*Thank you very much for taking the time to participate in this interview. Your responses have been very helpful and will help us to improve care for Veterans.*
