# Supplementary material for: Understanding the experience of veterans who require lower limb amputation in the veterans health administration
Source: PLoS One. 2022 Mar 18;17(3):e0265620. doi: 10.1371/journal.pone.0265620 (PMC8932557; doi:10.1371/journal.pone.0265620)
Supplement: Attachment — Submitted filename: Response to Reviewers.docx [file pone.0265620.s002.docx]

January 10, 2022

Editorial Board

PLOS One

**Manuscript**: Understanding the experience of Veterans who require dysvascular lower limb amputation in the Veterans Health Administration

Dear Editorial Board,

Thank you for the opportunity to revise our manuscript. We appreciate the editor and reviewer feedback and provide a point-by-point response below, detailing how we have addressed reviewer concerns. We feel that our revisions have strengthened the impact of the manuscript.

Role of Funder: This material is based upon work supported by the US Department of Veterans Affairs, Office of Research and Development, Rehabilitation Research and Development Grant number 1 I01 RX002960-01. The funders had no role in study design, data collection and analysis, decision to publish, or preparation of the manuscript.

Data availability: This paper reports on a qualitative study and excerpts of appropriate data are shared within the manuscript. We are unable to provide full de-identified interview transcripts as this was not discussed with participants during the consent process. Data requests can be addressed to the corresponding author.

We look forward to your feedback. Thank you for your time and consideration of our work.

Sincerely,

Chelsea Leonard, PhD

Investigator, Qualitative Methodologist, Seattle Denver Center of Innovation (COIN), Rocky Mountain Regional VA Medical Center, 1700 N Wheeling St, Aurora, CO 80045

p: 720-857-2787

e: [Chelsea.Leonard@VA.gov](mailto:Chelsea.Leonard@VA.gov)

| Critique | Response |
| --- | --- |
| Journal requirements: |  |
| 1. When submitting your revision, we need you to address these additional requirements. |  |
| Please ensure that your manuscript meets PLOS ONE's style requirements, including those for file naming. The PLOS ONE style templates can be found at | Thank you for this feedback. We have updated the formatting of the manuscript. |
| 2. Thank you for including your ethics statement:  "This study was approved by the local facility institutional review board: MIRB 01700; IRBNet # 1587998-12 |  |
| a. Please amend your current ethics statement to include the full name of the ethics committee/institutional review board(s) that approved your specific study. | Thank you for this comment, we have updated our ethics statement to include the name "Human Research Protection Program, VA Puget Sound Health Care System" |
| b. Once you have amended this/these statement(s) in the Methods section of the manuscript, please add the same text to the “Ethics Statement” field of the submission form (via “Edit Submission”). | We added this statement on the submission form. |
| 3. Thank you for stating the following financial disclosure: |  |
| “This material is based upon work supported by the US Department of Veterans Affairs, Office of Research and Development, Rehabilitation Research and Development Grant number 1 I01 RX002960-01.” |  |
| Please state what role the funders took in the study.  If the funders had no role, please state: ""The funders had no role in study design, data collection and analysis, decision to publish, or preparation of the manuscript."" | Thank you. The financial disclosure should read as follows: Role of Funder: This material is based upon work supported by the US Department of Veterans Affairs, Office of Research and Development, Rehabilitation Research and Development Grant number 1 I01 RX002960-01. The funders had no role in study design, data collection and analysis, decision to publish, or preparation of the manuscript. |
| Please include this amended Role of Funder statement in your cover letter; we will change the online submission form on your behalf. | Thank you. We included this in the cover letter. |
| 4. Thank you for stating the following in the Acknowledgments Section of your manuscript: |  |
| “This study was made possible by funding from the VA Office of Research and Development. The authors would like to gratefully acknowledge study participants for sharing their time and perspectives.” |  |
| We note that you have provided funding information that is not currently declared in your Funding Statement. However, funding information should not appear in the Acknowledgments section or other areas of your manuscript. We will only publish funding information present in the Funding Statement section of the online submission form. | Thank you. We have removed funding information from the Acknowledgements. |
| Please remove any funding-related text from the manuscript and let us know how you would like to update your Funding Statement. Currently, your Funding Statement reads as follows: | Thank you. We have removed funding related text from the manuscript. |
| “This material is based upon work supported by the US Department of Veterans Affairs, Office of Research and Development, Rehabilitation Research and Development Grant number 1 I01 RX002960-01.” |  |
| Please include your amended statements within your cover letter; we will change the online submission form on your behalf. | Thank you. We have included the following amended statement in the cover letter: This material is based upon work supported by the US Department of Veterans Affairs, Office of Research and Development, Rehabilitation Research and Development Grant number 1 I01 RX002960-01. The funders had no role in study design, data collection and analysis, decision to publish, or preparation of the manuscript. |
| [5. In your Data Availability statement, you have not specified where the minimal data set underlying the results described in your manuscript can be found. PLOS defines a study's minimal data set as the underlying data used to reach the conclusions drawn in the manuscript and any additional data required to replicate the reported study findings in their entirety. All PLOS journals require that the minimal data set be made fully available. For more information about our data policy, please see http://journals.plos.org/plosone/s/data-availability.](http://journals.plos.org/plosone/s/data-availability) | Thank you. We have included the following in our Data Availability Statement: This paper reports on a qualitative study and excerpts of appropriate data are shared within the manuscript. We are unable to provide full de-identified interview transcripts as this was not discussed with participants during the consent process. |
| [Upon re-submitting your revised manuscript, please upload your study’s minimal underlying data set as either Supporting Information files or to a stable, public repository and include the relevant URLs, DOIs, or accession numbers within your revised cover letter. For a list of acceptable repositories, please see http://journals.plos.org/plosone/s/data-availability#loc-recommended-repositories. Any potentially identifying patient information must be fully anonymized.](http://journals.plos.org/plosone/s/data-availability#loc-recommended-repositories) | We are unable to upload additional data, as per the data availability statement above. We meet the criteria for minimal dataset for qualitative data by sharing data excerpts within the manuscript. |
| [Important: If there are ethical or legal restrictions to sharing your data publicly, please explain these restrictions in detail. Please see our guidelines for more information on what we consider unacceptable restrictions to publicly sharing data: http://journals.plos.org/plosone/s/data-availability#loc-unacceptable-data-access-restrictions. Note that it is not acceptable for the authors to be the sole named individuals responsible for ensuring data access.](http://journals.plos.org/plosone/s/data-availability#loc-unacceptable-data-access-restrictions) |  |
| We will update your Data Availability statement to reflect the information you provide in your cover letter. |  |
| [6. We note that you have indicated that data from this study are available upon request. PLOS only allows data to be available upon request if there are legal or ethical restrictions on sharing data publicly. For more information on unacceptable data access restrictions, please see http://journals.plos.org/plosone/s/data-availability#loc-unacceptable-data-access-restrictions.](http://journals.plos.org/plosone/s/data-availability#loc-unacceptable-data-access-restrictions) | Thank you. We revised our data availability statement (please see above). |
| In your revised cover letter, please address the following prompts: |  |
| a) If there are ethical or legal restrictions on sharing a de-identified data set, please explain them in detail (e.g., data contain potentially sensitive information, data are owned by a third-party organization, etc.) and who has imposed them (e.g., an ethics committee). Please also provide contact information for a data access committee, ethics committee, or other institutional body to which data requests may be sent. | Thank you. We have added this information to our cover letter. |
| [b) If there are no restrictions, please upload the minimal anonymized data set necessary to replicate your study findings as either Supporting Information files or to a stable, public repository and provide us with the relevant URLs, DOIs, or accession numbers. For a list of acceptable repositories, please see http://journals.plos.org/plosone/s/data-availability#loc-recommended-repositories.](http://journals.plos.org/plosone/s/data-availability#loc-recommended-repositories) |  |
| We will update your Data Availability statement on your behalf to reflect the information you provide. |  |
| Please review your reference list to ensure that it is complete and correct. If you have cited papers that have been retracted, please include the rationale for doing so in the manuscript text, or remove these references and replace them with relevant current references. Any changes to the reference list should be mentioned in the rebuttal letter that accompanies your revised manuscript. If you need to cite a retracted article, indicate the article’s retracted status in the References list and also include a citation and full reference for the retraction notice. | Thank you. We have reviewed our references and are not making any changes. |
| Reviewers' comments: |  |
| **Comments to the Author** |  |
| 5. Review Comments to the Author |  |
| Reviewer #1: Surgeons performing the amputation may not have all the information as to rehab/prosthetic options. Would have been interesting to assess the surgeons' level of knowledge of life after surgery, because that may have affected their willingness to engage in meaningful discussions prior to amputation. | Thank you for this observation. We agree that it would be interesting to assess surgeons' level of knowledge of life after amputation and will address that question in future qualitative work. The goal of the current study was to understand patient experiences in their own words, and we did not talk to surgeons in this portion of the study. |
| Reviewer #2: This study is trying to explore the experience of Veterans during the amputation in VHA. A good engagement can usually promote the participation of patients with amputation. The insufficient shared decision making (SDM) during amputation is found in this study. To improve the benefits of amputees, this find is an important step for the development of a decision support tool and can help them engage the involvement. Although only twelve patients were recruited in this study, the results still showed an implication for an obstacle for the patients with CLTI to participate in the treatment of their own. This study is qualitative research and reveals some important points for further studies. However, more investigation should be done in the future to support the finding from this research due to the small sample size and limited sampling. | Thank you for this comment. We agree that the sample size of the current study was small, even for a qualitative study. We selected a small sample because this was an in-depth phenomenological study intended to inform a future larger qualitative study, and we are confident that we reached data saturation within our small sample. In the next phase of this study, we will follow up on our findings with a larger sample of patients. |
| There are several minor issues that need to be addressed before publication. |  |
| 1. The amputation usually changes the whole lifestyle of the people, sometimes it also makes the amputees under negative emotions. Most people with amputation feel sadness, madness, or other strong and negative emotions. Is SDM or involvement affected by undergoing these emotions? Could someone who is more optimistic feel they were involved more (or less) before surgery (in contrast, the subjects under sadness might feel more helpless or out of control)? | Thank you for this question. We are unable to address this question with the current dataset but hope to explore these ideas in another set of interviews with both patients and surgeons to learn how emotion affects their participation in SDM. |
| Did you observe any correlation between their responses or feels and the emotions of the subjects? | This is a good question. I have revised the second theme, "Actively working towards recovery as response to a perceived loss of independence" to reflect participants' emotional responses as this is where the analysts noted emotional response in the interviews. |
| 2. The authors mentioned that most subjects with diabetes perceive the amputation might happen to them. Most subjects in this study believe that the medical team would make the best choice and they don’t need to, or they couldn’t provide any personal thoughts on the discussion. Is there any possibility that the surgery should be done in rush time so made the medical team had no time to let the patients be involved in the decision-making? During the interview, had the subjects in this study ever mentioned or suggested that the debate on the amputation level could be done earlier? Would it be better if discuss with the patients with CLTI about the possible amputation level when the condition wasn’t going so badly? | This is a good observation. As shown in Table 2, most of the participants in this study received amputations in emergent settings, and it is very likely that this affected their experience with lack of SDM. In the subsequent stage of this study, we asked both patients and their surgeon's when amputation level conversations should take place. The goal of the current study was to understand participants' experiences more broadly, and the timing of amputation level conversations was not emergent in the interview data. |
| As the authors indicate in the study design, this research is a first but essential step before more extensive research to develop the patient decision aid and decision support tool. |  |
